# Development of stigma scale for women with mental illness in perinatal period, validity and reliability study

**DOI:** 10.1186/s12888-024-05523-7

**Published:** 2024-01-31

**Authors:** Esra Sen, Esra Yazici, Elif Kose, Yavuz Selim Ogur, Ahmet Bulent Yazici

**Affiliations:** 1 Department of Psychiatry, Medical Faculty, Sakarya University, Sakarya, Turkey; 2 Department of Public Health, Medical Faculty, Sakarya University, Sakarya, Turkey

**Keywords:** Perinatal, Psychiatric, Stigma scale, Validity and reliability

## Abstract

**Aim:**

Although there are many scales that measure stigma, there is no scale with the necessary adequacy to measure stigma in the perinatal period. The study aims to develop the stigma scale for women with mental illness in the perinatal period and test its validity and reliability.

**Materials and methods:**

Participants were reached via patients, visitors, and hospital staff who applied to Sakarya Training and Research Hospital between 01/06/2022 and 01/12/2022. Two hundred people (female *n* = 134, male *n* = 66) aged 18–65 participated in the study and "Sociodemographic data form," "Perinatal Mental Illness Stigma Scale (PMISS)," "Social Distance Scale," and "Beliefs Towards Mental Illness Scale" were used to collect data. Data were analyzed using SPSS 22 and the AMOS 26 program.

**Results:**

The Content Validity Index of the scale items was between 0.80–1. Cronbach's alpha coefficient score of the general scale was 0.94, the "Discrimination and Prejudice" sub-dimension was 0.93, and the "Labeling" sub-dimension was 0.88. It was determined that item-total score correlations varied between 0.410 and 0.799. P value calculated < 0.05 in Barlett's test and 0.94 in the Kaiser-Meyer Olkin test. These values show that factor analysis can be applied to the scale. According to the Exploratory Factor Analysis result, the scale has a 2-factor structure, explaining 60% of the total variance. The Guttman Split-Half coefficient of the scale was 0.882, and the Spearman-Brown coefficient was 0.883. The scale was reapplied to 30 participants with an interval of three weeks. The correlation coefficient between the two measurements was 0.91, indicating that the scale satisfies the invariance principle over time.

**Conclusion:**

The PMISS is a reliable measurement tool that can be used to investigate stigma towards mental illness during the perinatal period in the Turkish population.

**Supplementary Information:**

The online version contains supplementary material available at 10.1186/s12888-024-05523-7.

## Introduction

Stigma is a negative attitude that society develops towards mental disorders and some illnesses, leading to the person's exclusion. Prejudices, which are negative attitudes or opinions towards people or some groups, create social distance towards these people. People with mental illness have been stigmatized many times from the past to the present. Society has seen mental illness as a sign of deviance, personal inadequacy, low intelligence, weakness, incompetence, or unreliability. People with mental illness have also been judged to be violent and unpredictable. Such negative attitudes and behaviors can be found in many parts of society, particularly in people's families, social circles, and among health professionals. Nor does stigma vary much between countries and regions [1].

Stigmatization serves as a deterrent for help seeking for numerous individuals with mental disorders, leading to a consequential cessation or abstention from treatment-seeking endeavors. In this context, it is concluded that stigma inflicts harm upon individuals with mental illness and, by extension, upon society at large, engendering inequitable circumstances and deleterious consequences [1, 2]. In a study of major depressive disorder, stigma was associated with treatment incompliance, negative emotional experiences, and decreased self-esteem [3]. Another study reported that self-stigma is related to low self-efficacy and low self-esteem, that self-stigma results from social stigma, and that studies should be conducted to increase public awareness to benefit patients [4].

Exposure to stigma also affects people's mental health in the perinatal period. Identifying stigma in the perinatal period is essential for the mother's mental health and the baby's healthy development. There have been many studies on stigma, but studies on stigma in the perinatal period are limited. So far, a perinatal internal stigma has been developed, which assesses the internal stigma of women with perinatal mental illness, but it has not yet been translated into our language [5]. To the best of our knowledge, no scale measures the social stigma of mental illness in the perinatal period. This study aims to develop a scale that can be used by women with mental illness in the perinatal period but also by healthy people and, therefore, by a large population. This study aims to develop the Perinatal Mental Illness Stigma Scale and test its validity and reliability.

## Materials and method

### Place and time of research

This study was conducted with 200 participants from the community. Participants were reached via patients, relatives, and hospital staff who applied to Sakarya Training and Research Hospital between 01/06/2022 and 01/12/2022.

### Sample of the research

Reviewing the literature found that the sample size was calculated on scale items and variables in scale development studies. The literature states that a sample of 100–200 people is sufficient, especially if the factors are clear and robust and the number of variables is small [6]. It is generally accepted that the sample size should be at least five or even ten times the number of variables [7]. As the final version of the scale consisted of 20 items, the sample size was 200 (20 × 10 = 200) people.

The sample was randomly selected from participants aged 18–65 years with a similar distribution in terms of different genders, socio-economic levels, education levels, and employment status were included in the study. To ensure that the participants reflected all of society, healthy and ill people were included in the study, except those with diseases known to affect cognitive function, willpower, and judgment.

### Stages of the study

#### Creation of scale items

The first form of the Perinatal Mental Illness Stigma Scale (PMISS) was created with 40 questions by conducting a literature review on the scales developed on stigmatization related to mental illnesses, factors related to stigma, perinatal diseases, and motherhood skills. Twenty items, which were thought to reflect perinatal stigma better, were selected from the 40-item scale by taking the opinion of a team of 20 faculty members and psychiatry residents working in the field of psychiatry.

#### Getting expert opinion

**Fig. 1:**
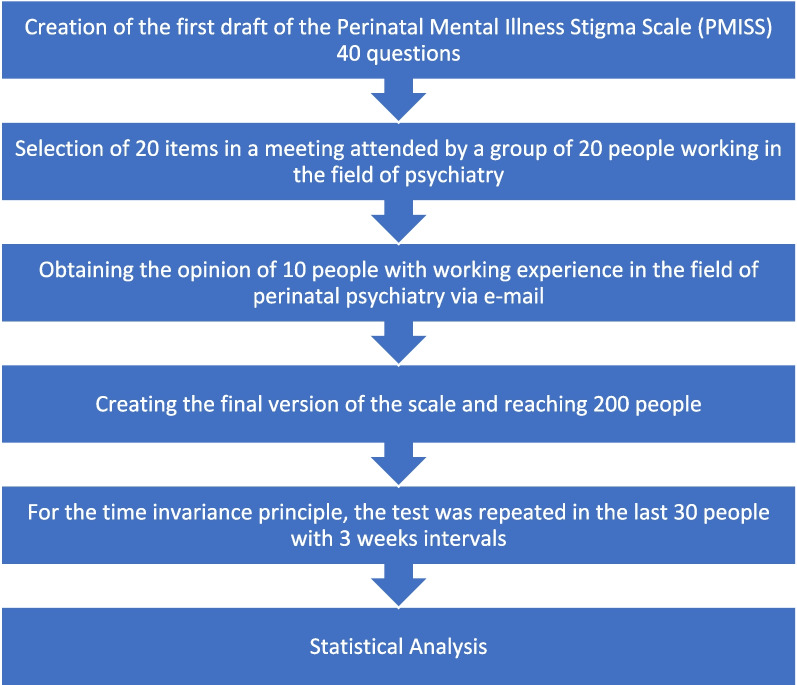
Summary of implementation phases

For content validity, the 20-item scale was sent via e-mail to a team of 10 experts working in the field of perinatal mental health. After the expert opinions, the content of the 8th, 17th, 18th and 20th items were adjusted. After the expert opinion, the 20-item scale was finalized and the application part was started. A summary of the implementation stages is given in Fig. 1.

### Data collection tools

#### Sociodemographic data form

It is the form filled by the participants, which includes the sociodemographic characteristics of the patients participating in the study, such as age, education level, marital status, socioeconomic level, and the history of mental illness and additional medical illness.

#### Perinatal Mental Illness Stigma Scale (PMISS)

After the expert opinion, the final version of the scale was created as 20 items. The scale aims to measure the opinions of other people about women with psychiatric illness in the perinatal period. The high score obtained from the scale indicates that the stigmatization of women with mental illness in the perinatal period is high. The scale has a 5-point Likert-type rating as "I totally disagree", "I partially disagree", "I neither agree nor disagree", "I partially agree", "I totally agree". Filled in by the participants.

#### Social Distance Scale (SDS)

While developing the scale, well-known Star sample cases describing paranoid schizophrenia and anxiety neurosis/depression were presented to the participants, and a psychiatric diagnosis was not written to the cases. Each subject responded to only one randomly selected sample case. After the sample case, a 14-question scale developed by the researcher was applied. This scale aims to measure the desired social distance between the mentally ill and the person in order to examine the social rejection of the mentally ill in our society. Findings showed that there is more acceptance of being in impersonal environments with the people depicted in the examples, and less desire to interact with these people in environments involving social intimacy. In our study, there was no star case sample, and it was stated that the form should be filled considering women with mental illness. The validity and reliability study of the scale was carried out by Haluk Arkar in 1991 [8].

#### Beliefs Toward Mental Illness Scale (BMI)

It is a scale whose validity and reliability study was completed in 2008, which was created to determine the positive and negative beliefs of individuals with different cultural characteristics about mental illnesses. The final version of the 21-item scale consists of three sub-scales: "Dangerous", "Helplessness", "Poor Social and Interpersonal Skills" and is a 6-point Likert type. Although the scale is interpreted on both subscale scores and scale total scores, a high score indicates negative belief in mental illnesses [9].

### Validity and reliability of the perinatal mental illness stigma scale

#### Content validity

After the 20-item version of the scale was created, it was presented to the expert opinion of 10 people with experience working in the field of perinatal mental health, and the answers were evaluated with the Davis technique. They were asked to answer the questions as "A = The item is appropriate, B = The item should be edited lightly, C = The item should be edited seriously, D = The item is not appropriate." According to the Davis technique, The ratio of those who chose A (item appropriate) and B (item should be slightly modified) to the total number of experts constitutes the Content Validity Index (CVI), and this value should be 0.80 and above [10].

#### Construct validity

Factor analyses are used for the construct validity of the scale. Before factor analysis, it is necessary to test the sample size's adequacy and the data's suitability for factor analysis. Exploratory factor analysis** (**EFA) and Confirmatory Factor Analysis (CFA) were used for the construct validity of the scale. Rotation is used to simplify the structure of the factors. Varimax and oblimin techniques were used for rotation [11]. The suitability of the sample size was examined using the Kaiser–Meyer–Olkin (KMO) value. For the sample size to be sufficient, the KMO must be greater than 0.50 [12]. Another study stated that the KMO value should be above 0.60 to perform factor analysis [13].

Before performing factor analysis, it is necessary to check whether the correlation matrix is appropriate. The suitability of the correlation matrix is evaluated with Bartlett's Sphericity test. Bartlett's Sphericity test tests the hypothesis that the correlation matrix is the unit matrix. P values < 0.05 in Barlett's test indicate the suitability of the scale for factor analysis.

#### Internal consistency

The internal consistency of the items in the scale was evaluated with the Cronbach Alpha Coefficient, splitting the test in half, the item-total score correlation, and the comparison of the 27% lower and upper slices of the scale total score. Item-total score correlations are another internal consistency measure.

Item-total score correlation evaluates the relationship between a scale item and the total score of the scale items. It is used to determine how accurately the item measures the trait being measured. If the item-total score correlation is high, the item is highly discriminating. If the correlation coefficient is low, it can be said that the item is not reliable enough, and its discrimination is low [14]. It has been suggested that the item-total correlation should be greater than 0.20 for each item [15].

#### Summability and confirmatory factor analysis

Tukey's summability test was used to assess whether the scale is an additive scale type. Structural equation modeling (SEM) is an analysis method that combines multivariate regression analysis and factor analysis. With SEM, the compatibility of the statements that are claimed to exist theoretically, and thus their verification, is carried out [16]. CFA is an example of SEM that deals with the relationships between observed measurements and latent variables or factors. Measures observed with CFA are interrelated and variables or factors affect correlations between observations [17]. The fact that the fit index values calculated during CFA are within the recommended range indicates that the developed hypothesis is correct [18].In the SEM, CMIN/df values of fit index < 3 indicates ideal result, < 5 indicates acceptable fit, > 0.90 good fit for NFI, CFI, IFI, TLI, < 0.05 ideal result for RMSEA (< 0.08 good fit, < 0.10 indicates acceptable fit) [19].

#### Invariance of scale over time

Invariance over time was measured by the test–retest method. The fact that the results of two measurements made at different times are similar indicates that the scale is invariant over time. For the test–retest process, it is stated that the scale should be re-administered to at least 30 people with an interval of 2–4 weeks [20]. Pearson correlation value greater than 0.7 between two measurements indicates a high correlation [21].

### Examining the data

The data of the research were analyzed by IBM SPSS 22 and IBM AMOS 26 program. A free trial version of IBM AMOS 26 was used. Normality distribution of the data was examined with the Kolmogorov–Smirnov test and Z test. The statistical methods used in the study are shown in Table 1 in detail.

**Table 1:** Statistical methods used in the research

Examined feature	Statistical methods
Normality distribution of data	Z test, Kolmogorov Smirnov test
**Validity**	
Content validity of the draft scale	Davis Technique, evaluation of expert opinions
Criterion validity	Correlation with a similar scale
Construct validity	Exploratory factor analysis (EFA)
Sample size and suitability tests for factor analysis	Kaiser–Meyer–Olkin (KMO) sample adequacy test Bartlett's Test of Sphericity
Construct validity of draft scale sub-dimensions	Confirmatory Factor Analysis (CFA)
**Reliability**	
Internal consistency	Cronbach Alpha Coefficient, split-half technique, item-total score correlation, correlation between items, comparison of 27% lower and upper slices of the scale total score
Invariance over time	Test–retest method, Wilcoxon paired-sample test
Splitting the test in half	Spearman Brown test
**Other analyzes related to scale score**	
Determining the sociodemographic characteristics of the participants	Number, percentage, mean and standard deviation
The relationship between the sociodemographic characteristics of the participants and the scale total score	Independent groups t test, ANOVA test, Kruskal Wallis test
Examining the relationship between the sub-dimensions of the scale and testing the significance	Pearson product-moment correlation analysis, Point scatter plots

## Results

### Sociodemographic and clinical features

The Perinatal Mental Illness Stigma Scale (PMISS) study involved 200 participants, 66 (33%) of whom were male and 134 (67%) were female. The participants' mean age was 34.68 (minimum 18, maximum 65, median 31, mean 34.68). Table 2 provides the sociodemographic characteristics of the participants.

**Table 2:** Sociodemographic characteristics of the PMISS study participants

Sociodemographic Characteristics		Patient Data
**Age Distribution (years)**	Minimum	18
	Maximum	65
	Mean	34,68 ± 12,24
**Gender**	Female	%67 (*n* = 134)
	Male	%33 (*n* = 66)
**Education level**	Primary Education Graduate	%17,5 (*n* = 35)
	High school graduate	%21,5 (*n* = 43)
	University Graduate	%51 (*n* = 102)
	Post Graduate	%10 (*n* = 20)
**Marital Status**	Married	%55 (*n* = 110)
	Single	%41 (*n* = 82)
	Widow	%2 (*n* = 4)
	Divorced	%2 (*n* = 4)
**Status of Having a Child**	Yes	%46 (*n* = 92)
	No	%54 (*n* = 108)
**People Living With**	Alone	%14 (*n* = 28)
	Spouse/family	%79 (*n* = 158)
	Friend	%4 (*n* = 8)
	Other (dormitory, nursing home, etc.)	%3 (*n* = 6)
**Living place**	City center	%85,5 (*n* = 169)
	Rural	%15,5 (*n* = 31)
**Working Status**	Student	%15,5 (*n* = 31)
	Unemployed	%20 (*n* = 40)
	Works at an irregular job	%4 (*n* = 8)
	Works at a regular job	%58 (*n* = 116)
	Retired	%2,5 (*n* = 5)
**Economic Level**	0–5.000 TL^a^	%10,5 (*n* = 21)
	5.000–10.000 TL	%26,5 (*n* = 53)
	10.000–15.000 TL	%22 (*n* = 44)
	15.000–20.000 TL	%13 (*n* = 26)
	20.000–25.000 TL	%6 (*n* = 12)
	25.000 TL and above	%22 (*n* = 44)

^a^The net minimum wage in Turkey at the time of the research was 5500 TL

When mental history of the participants evaluated it is seen that; thirty-seven people (18.5%) were diagnosed with a mental illness, and 163 (81.5%) were not. Thirty-five people (94.5%) diagnosed with a psychiatric disorder received treatment, while two (5.5%) did not. Looking at the treatment distribution, 30 people (85.8%) received outpatient medication, 3 (8.5%) received both outpatient medication and psychotherapy, and 2 (5.7%) received psychotherapy only. None had ever received inpatient treatment or ECT.

When considering whether people had a psychiatric illness in their family or close environment, 39% (*n* = 78) had someone diagnosed with a psychiatric illness in their immediate environment, and 61% (*n* = 122) did not.

### Findings related to the validity and reliability of the perinatal mental illness stigma scale

#### Content validity

Content validity was assessed with the Davis technique. The content validity index of the PMISS items was between 0.80–1. According to the experts' suggestions, the contents of the 8th, 17th, 18th, and 20th items were revised, and the total number of items did not change.

#### Internal consistency

The Cronbach alpha coefficient of the scale was found to be 0.949. When the calculations were made by removing the items, it was found that the Cronbach alpha coefficient of the scale varied between 0.945 and 0.951 (Table 3). As there was no significant change, no item was removed from the scale. The Cronbach's alpha coefficients of the PMISS sub-dimensions were calculated separately. The sub-dimension 'Discrimination and Prejudice (Factor 1)' was found to be 0.93, and the sub-dimension 'Labelling (Factor 2)' was 0.88.

**Table 3:** Item total scale score statistics of PMISS Cronbach alpha coefficient

Question Number	Mean	Standard deviation	Scale mean^a^	Scale variance^a^	AI and TS ^b^ Correlation	CAC of the scale^a^
**Question 1**	3,45	1,40	53,63	337,521	0,557	0,948
**Question 2**	2,40	1,47	54,67	339,768	0,503	0,950
**Question 3**	3,03	1,49	54,04	329,420	0,694	0,946
**Question 4**	3,97	1,08	53,11	348,742	0,474	0,949
**Question 5**	3,62	1,32	53,45	334,681	0,676	0,947
**Question 6**	3,34	1,41	53,73	328,005	0,762	0,945
**Question 7**	3,27	1,36	53,81	327,994	0,795	0,945
**Question 8**	2,60	1,37	54,47	346,401	0,410	0,951
**Question 9**	2,62	1,42	54,46	329,124	0,737	0,946
**Question 10**	2,88	1,27	54,19	334,469	0,709	0,946
**Question 11**	3,13	1,30	53,95	331,997	0,744	0,946
**Question 12**	2,94	1,34	54,13	329,102	0,784	0,945
**Question 13**	2,34	1,33	54,73	332,326	0,723	0,946
**Question 14**	2,34	1,32	54,74	333,902	0,691	0,946
**Question 15**	2,54	1,26	54,54	333,878	0,728	0,946
**Question 16**	2,43	1,28	54,65	335,173	0,688	0,947
**Question 17**	2,78	1,34	54,29	328,551	0,799	0,945
**Question 18**	2,67	1,31	54,40	330,574	0,769	0,945
**Question 19**	2,10	1,40	54,98	337,527	0,574	0,948
**Question 20**	2,58	1,31	54,50	332,714	0,724	0,946
**SCALE**	**Mean**	**Variance**	**Standard deviation**	**Number of questions**	**Cronbach’s Alfa**	**Range**
	57,08	369,200	19,21457	20	0,949	20

*CAC* Cronbach Alpha Coefficient

^a^When item was deleted

^b^Adjusted items and total score

The item-total correlations varied between 0.410 and 0.799; the results were significant at *p* < 0.001.

The Cronbach's alpha coefficient was calculated separately for the test's first part (ten questions) and the second part (ten questions). This value was 0.894 for the first part of the scale and 0.935 for the second part; the Guttman Split-Half coefficient was 0.882, and the Spearman-Brown coefficient was 0.883.

In assessing internal consistency, the total scores obtained from the scale are ordered from highest to lowest, and the relationship between the lower and upper 27% is examined. The difference between the two groups was statistically significant (*p* < 0.001).

#### Construct validity

There is no reverse item in the scale, the correlation between scale items was found to be 0.211 at the lowest and 0.791 at the highest. The suitability of the sample size was examined using the KMO value. The KMO value was found to be 0.945 and sample size accepted a sufficient.

Chi Square = 2794,639, *p* < 0.001 was found in the Bartlett's Sphericity test. The result obtained shows that the correlation matrix is suitable and factor analysis can be applied to the data.

Varimax and Oblimin vertical rotation were applied to the data. Since three factors combined after varimax vertical rotation and six factors combined after oblimin, the data obtained with the oblimin technique were used. As a result of EFA, no item was removed from the scale.

As a result of Principal Component Analysis, it was found that the scale consisted of two factors. The items constituting the 2-factor structure of PMISS were examined; first factor consists of 8th, 9th, 10th, 11th, 12th, 13th, 14th, 15th, 16th, 17th, 18th, 19th, 20th items. The first sub-dimension is called “Discrimination and Prejudice”. The second factor consists of 1st, 2nd, 3rd, 4th, 5th, 6th, and 7th items, and this sub-dimension is called “Labeling.”

As a result of applying the oblimin rotation technique, it resulted in a 2-factor structure with an eigenvalue above 1.00 and 60.48% of the total variance.

**Fig. 2:**
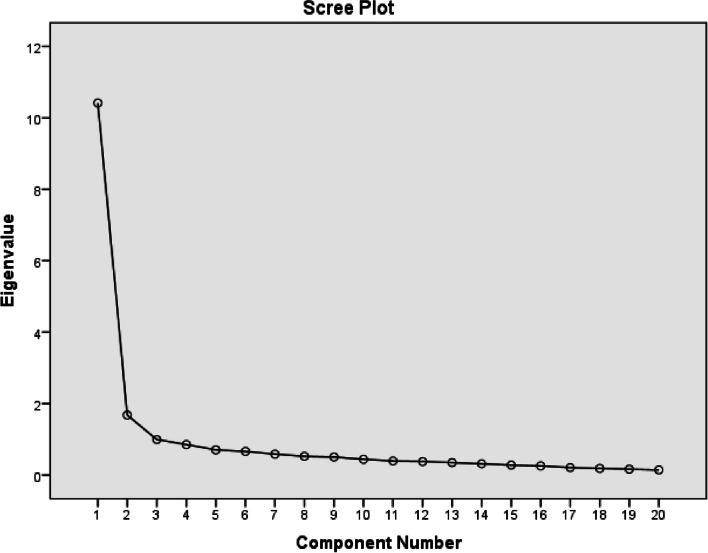
Scree plot chart

In the graph created with the Scree Plot test, the number of factors is determined by looking at how many factors exist until the first sharp change. Since the first sharp change in the graph was in the 2nd factor, the factor number was accepted as 2, in line with the previously determined factor. (Fig. 2.).

#### Summability and confirmatory factor analysis

According to the result of the analysis of variance with Tukey's summability test, the scale is collectible. (Nonadditivity: F = 1,571, *P* = 0,210 > 0,05).

After examining whether the data are normally distributed, CFA applied. (Kolmogorov–Smirnov value was 0.043 and *p* = 0.200).

**Fig. 3:**
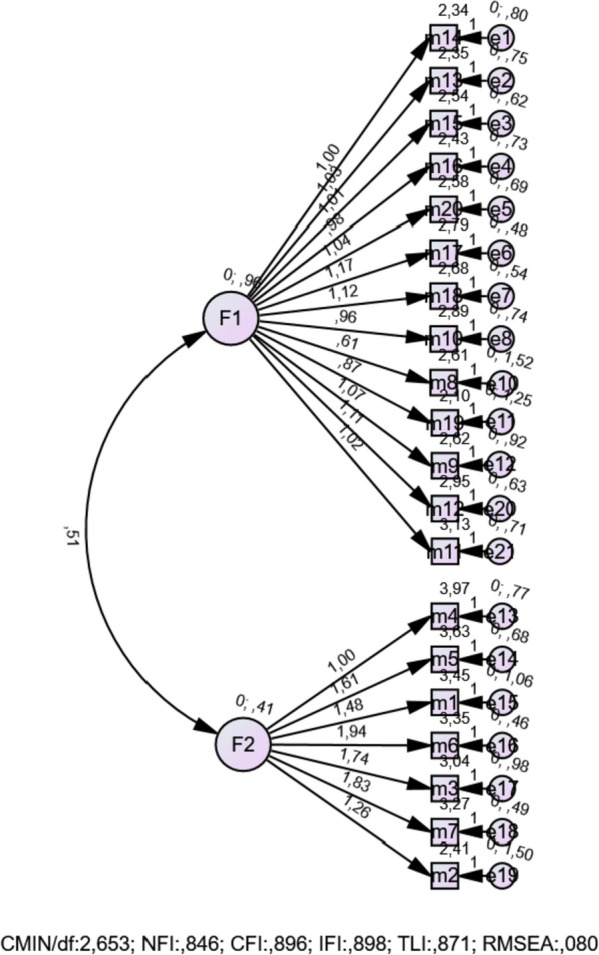
First-level CFA results

Structural equation modeling (SEM) results examined by CFA were found to be in acceptable fit. (*P* < 0.001, X2/df = 2,653, RMSEA = 0,080, NFI = 0,846, CFI = 0,896, IFI = 0,898, TLI = 0,871, AIC = 570,396) First-level CFA values and fit indices in our study are given in Fig. 3.

#### Correlation with other scales

For the correlation of the developed scale, the responses given to the Social Distance Scale (SDS) and the Beliefs Toward Mental Illness Scale (BMI) whose validity and reliability were proven in terms of stigma, were compared with the Pearson correlation coefficient. A positive correlation was found between the responses of the participants to the scales. The correlation of PMISS with BMI was 0.765, and its correlation with SDS was 0.640 (Table 4).

**Table 4:** Correlation of scale scores

**Scale items total correlation**		**PMISS**	**BMI**	**SDS**
PMISS	r p	1	0,765 < 0.001	0,640 < 0.001
BMI	r p	0,765 < 0.001	1	0,650 < 0.001
SDS	r p	0,640 < 0.001	0,650 < 0.001	1

#### Invariance of scale over time

The scale was reapplied to 30 participants three weeks apart. The correlation coefficient between measurements was 0.91, indicating that the scale satisfies the principle of invariance over time.

## Discussion

The study's results indicate that the PMISS is a valid scale for measuring perinatal stigma towards mental illnesses. The scale demonstrates internal consistency, construct validity, and consistent results in retest measurements. This research is essential as it is the first scale study to validly and reliably measure the social stigmatization of mental illnesses seen in perinatal women.

The validity and reliability analysis of the scale first started with internal consistency analysis. The internal consistency of the scale is defined as the items being compatible with each other and the entire scale and measuring the same structure in a consistent manner [22]. In our study, the Cronbach Alpha coefficient of the general scale was found to be 0.94, the factor 1 sub-dimension was 0.93, and the factor 2 sub-dimension was 0.88. A value of 0.7 or higher showed adequate reliability, while a value of 0.8 or higher showed good internal consistency [23, 24].

Item-total score correlations are another measure of internal consistency. In item-total score correlation, the relationship between a scale item and the total score of the scale items is evaluated. Item-total score correlation is used to determine how accurately the item measures the feature it is intended to measure. When the item-total score correlation is high, the discrimination of that item is high; when the correlation coefficient is low, it can be said that the item is not reliable enough and its discrimination is low [14]. It was stated that the item-total score correlation in the developed scales should be over 0.20 and there should be no negative correlation between the items [14]. There are no reverse items in the scale, the lowest item-total score correlation was 0.410 and the highest was 0.799. The correlation between the scale items was found to be the lowest 0.211 and the highest 0.791. The fact that the correlation between the scale items and the total score is above 0.20, that the scale does not contain reverse items, and that no negative correlation is detected between the items shows that the PMISS does not contain any problematic items and provides sufficient internal consistency criteria.

Another method used for internal consistency analysis is the calculation of Cronbach's Alpha reliability coefficient after splitting the test in half technique. Two separate groups were created, consisting of the first 10 questions and the second 10 questions. This value was found to be 0.894 for the first part of the scale and 0.935 for the second part. The Guttman Split-Half coefficient of the scale was found to be 0.882 and the Spearman-Brown coefficient was 0.883, and the correlation between the two parts was found to be 0.790. Since values above 0.70 are considered reliable in the literature, these findings show that the internal consistency reliability of PMISS is high [6, 23, 24].

In scale development studies, after internal consistency analyzes are completed, construct validity analyzes should also be performed. It has been reported in the literature that before factor analysis, the adequacy of the size of the sample group for factor analysis should be tested. In factor analysis, it is determined whether the size of the sample group is sufficient or not by using the KMO test [25]. In order to apply factor analysis, it is recommended that the KMO value be over 0.60. In the literature, KMO of 0.90–1 is accepted as an excellent reliability value, but in our study, the KMO value was found to be 0.945, that is, in the perfect range. The KMO value being determined as 0.945 shows that sufficient sample group size was provided for factor analysis. Before factor analysis, it is necessary to check whether the correlation matrix is appropriate. Whether the correlation matrix is appropriate or not is evaluated by Bartlett's Sphericity test. With Bartlett's Sphericity test, the hypothesis that the correlation matrix is an identity matrix is tested. In our study, Chi Square = 2794.639, *p* < 0.001. The result obtained shows that the correlation matrix is appropriate and factor analysis can be applied to the data [26–29].

The second stage of the EFA application involves examining the factor structure of the scale. It is recommended to use Principal Component analysis and rotation techniques to determine the factor structure of the scale. As a result of the application of these analyzes in our study, a 2-factor structure with an eigenvalue above 1.00 was obtained, explaining 60.48% of the total variance. It is necessary to evaluate whether the factor structure can be reduced with the scree plot test. In the graph created by the Scree Plot test, the number of factors is decided by looking at how many factors there are until the first sharp change. Since the first sharp change in the graph was in the 2nd factor, the number of factors was accepted as 2. Factor analysis was repeated after oblimin rotation application. A 2-subdimensional scale with an eigenvalue over 1.00 was obtained, explaining 60.48% of the total variance in PMISS. It was found that 34.729% of the total variance of 60.48% in the study was explained by the "Discrimination and Prejudice" factor and 25.752% by the "Labeling" factor. It is stated in the literature that the variance of the scale should be between 40–60% [30]. In our study, a total variance of 60.48% was obtained and was found to be sufficient.

In the final stage of EFA, factors are named by looking at the content of the items. This naming process is called "labeling". In our study, by looking at the items in the factors, the first factor was named "Prejudice and Discrimination" and the second factor was named "Labeling". Factor 1's higher stigma burden and behavioral components led it to be named "Prejudice and Discrimination". Despite this, the term "Labeling", which emerged as the first component of stigma, was deemed appropriate since the 2nd Factor contains negative expressions related to stigma, but contains more accepting and less certain approaches. The statement "If we were to explain the term stigma on a level plane, the first step would be labeling and the last step would be discrimination", mentioned in an article, guided us in naming [2].

In scale development studies, it is recommended to perform CFA after EFA. CFA provides strong evidence to support the validity of the factor structure of a measure [18]. The accuracy of the hypotheses obtained with CFA is tested [31]. CFA should be applied after examining whether the data is normally distributed or not. In our study, the normality distribution of the data was examined with the Kolmogorov–Smirnov test. P value was found to be 0.200, *p* > 0.05, and the data show normal distribution.

Structural equation modeling (SEM) is an analysis method that combines multivariate regression analysis and factor analysis. With SEM, statements that are hypothesized to exist theoretically are verified [16]. CFA is an example of a type of SEM that deals with the relationships between observed measurements and latent variables or factors. The fact that the fit index values calculated during CFA are within the recommended range indicates that the developed hypothesis is correct [18]. In SEM fit index values, < 3 for CMIN/df indicates ideal result, < 5 indicates acceptable fit; For NFI, CFI, IFI, TLI, > 0.90 indicates good fit; For RMSEA, < 0.05 indicates ideal result, < 0.08 indicates good fit, < 0.10 indicates acceptable fit [19]. In our study, CMIN/df was calculated as 2.653, NFI 0.846, CFI 0.896, IFI 0.898, TLI 0.871, AIC 570.396, RMSEA 0.080, and since these values are within the desired range, they indicate acceptable fit. As a result of the first level CFA results and fit indices, no items were removed from the scale.

For the correlation of the developed scale, the responses given to the SDS and BMI, whose validity and reliability have been proven regarding stigma, were compared with the Pearson correlation coefficient. A positive correlation was found between the participants' responses to the scales. The positive correlation between the three scales indicates that as the scores from the scales increase, the level of stigmatization increases and similar results occur.

Invariance over time was measured by the test–retest method. The fact that the results of two measurements made at different times are similar indicates that the scale is invariant over time. It is stated that for the test–retest method, the scale should be re-applied to at least 30 people with an interval of 2–4 weeks [20]. It is recommended that the Pearson correlation coefficient between two measurements be over 0.70 [21]. In accordance with the literature, the test was repeated on 30 volunteers every 3 weeks. The correlation coefficient between the measurements was found to be 0.916. This value shows that the principle of invariance against time is met.

## Conclusion

As a result, it has been determined that PMISS is a valid and reliable measurement tool in determining the stigmatization attitudes of Turkish society towards women with mental illness in the perinatal period. Our study was conducted in a heterogeneous group, and the stigma level of homogeneous subgroups may also be the subject of research. With the scale we developed, stigmatization towards others, that is, social stigma, was measured. Social stigma is an important indicator of internalized stigma, and different scales examining internal stigma may also need to be developed.

### Supplementary Information

**Additional file 1: **Perinatal Mental Illness Stigma Scale (PMISS).

## Data Availability

The datasets used and/or analysed during the current study are available from the corresponding author on reasonable request.
