# Supplementary figures and images for: Development of stigma scale for women with mental illness in perinatal period, validity and reliability study
Source: BMC Psychiatry. 2024 Jan 31;24:89. doi: 10.1186/s12888-024-05523-7 (PMC10832070; doi:10.1186/s12888-024-05523-7)

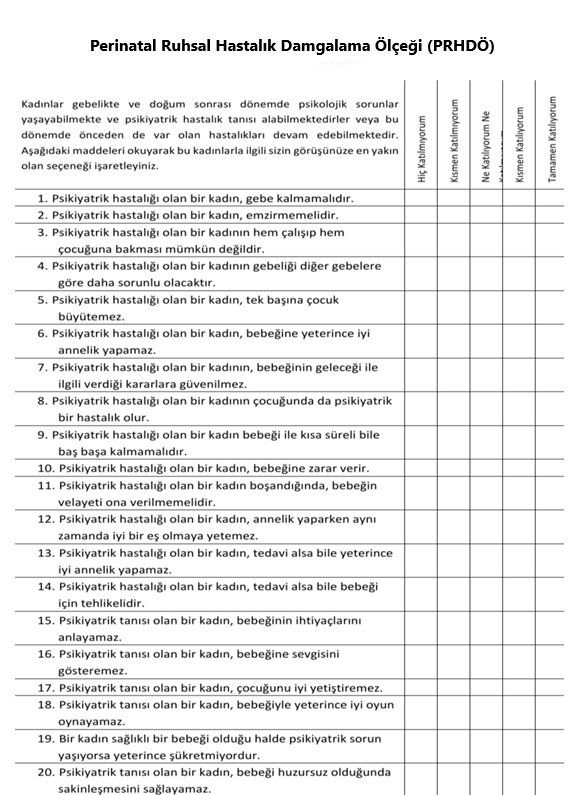

Supplement: Supplementary file 1 — Additional file 1: Perinatal Mental Illness Stigma Scale (PMISS). [file 12888_2024_5523_MOESM1_ESM.png]
